# Real-world implementation of a standardized ICU protocol for daytime-restricted enteral nutrition in critically ill adults: A retrospective quality improvement study

**DOI:** 10.1016/j.nutos.2025.100622

**Published:** 2026-01-09

**Authors:** Kelsey Russell-Murray, Hassan S. Dashti

**Affiliations:** a St. Thomas Elgin General Hospital, St. Thomas, ON, Canada; b Department of Anesthesia, Critical Care and Pain Medicine, Massachusetts General Hospital, Boston, MA, USA; c Division of Sleep Medicine, Harvard Medical School, Boston, MA, USA; d Division of Nutrition, Harvard Medical School, Boston, MA, USA

**Keywords:** Enteral nutrition, Intensive care unit, Critically ill adults, Chronobiology, Gastrointestinal intolerance

## Abstract

**Background:**

Enteral nutrition (EN) delivery is often interrupted in the intensive care unit (ICU), and while continuous 24-hour feeding is standard practice, emerging evidence from circadian biology and pilot trials suggests that daytime-restricted EN may enhance nutritional adequacy and patient outcomes by aligning feeding with biological rhythms.

**Methods:**

This quality improvement study describes a novel, standardized daytime-restricted EN protocol in a community hospital ICU and retrospectively evaluate its real-world implementation. The protocol involved a stepwise transition in EN delivery, beginning with continuous trophic feeding (acute/initial phase), followed by daytime-restricted 12-hour cyclic feeding (anabolic recovery phase), and advancing to intermittent daytime-restricted feeding (chronic recovery phase). A convenience sample of 22 adult ICU patients (12 received continuous 24-hour EN; 10 with the daytime-restricted EN protocol) was analyzed. Clinical data were extracted from electronic medical records, including EN infusion rates, duration, and interruptions.

**Results:**

Patients in the daytime-restricted group received EN at higher infusion rates (median 87.5 vs. 40.0 mL/hr), over fewer hours per day (11.0 vs. 14.5 hours), experienced fewer interruptions (1.0 vs. 9.5 hours/day), and received a greater percentage of their prescribed nutritional volume (90.0% vs. 57.5%) compared to the continuous group (all *P* value < 0.05). Vomiting was more frequently reported in the daytime-restricted group, while constipation was more common in the continuous group, though these differences were not statistically significant.

**Conclusion:**

This preliminary evaluation supports the feasibility of implementing a daytime-restricted EN protocol in an adult ICU and suggests potential advantages in delivery consistency and nutritional adequacy. To support broader implementation, larger prospective studies across broader ICU populations are necessary.

## Introduction

Enteral nutrition (EN) is vital for supporting critically ill patients unable to maintain adequate oral intake by providing calories and essential nutrients to prevent malnutrition, promote recovery, reduce infection risk, and improve clinical outcomes. [1–5] However, EN is frequently interrupted in intensive care units (ICUs), contributing to underfeeding, delayed recovery, and increased risk of morbidity and mortality. [6] Studies indicate that over 30% of patients experience interruptions to their EN and that EN is disrupted on nearly 50% of ICU days, most often due to gastrointestinal intolerance (e.g., abdominal distension, diarrhea, high gastric residual volumes, vomiting), procedural delays, airway management, or tube-related complications. [6–10] Feedings are frequently withheld for 5–7 hours per day, resulting in patients receiving only about 60% of their prescribed nutritional goals. [7] Therefore, novel strategies for EN delivery in the ICU are essential to limit underfeeding.

Although continuous 24-hour EN remains the clinical standard in ICUs, evidence supporting its safety and efficacy remains limited. [11] Continuous EN is presumed to reduce the risk of aspiration and other related complications by delivering nutrients more slowly than cyclic or intermittent feeding schedules. [7] Emerging evidence from circadian biology suggests that aligning EN with the body’s endogenous circadian rhythms through daytime feeding may confer metabolic, gastrointestinal, functional, and immune benefits, while continuous feeding may contribute to impaired metabolism, circadian disruption, poor sleep, and increased insulin resistance; relevant trials have been summarized in prior reviews. [12,13] The growing evidence supports the use of daytime, time-restricted nutrition support, as we recently outlined. [12].

To date, only a few controlled trials have evaluated daytime-restricted nutrition support, and no standardized protocols or real-world evidence on tolerance are available. [12] Such data are essential to support broader implementation. The aim of this quality improvement study was to describe a novel, standardized daytime-restricted EN protocol implemented in a community hospital ICU and to retrospectively evaluate its real-world application.

## Materials and methods

### Study design

The study was a retrospective quality improvement evaluation of a novel daytime-restricted EN protocol implemented in a Level 3 ICU at a community hospital to assess its feasibility and tolerance in a real-world setting. The St. Thomas Elgin General Hospital is a 157-bed community facility serving St. Thomas and Elgin County in Ontario, Canada, with a 14-bed, closed Level 3 ICU that provides advanced respiratory support and continuous monitoring for critically ill adult patients. The analysis was approved by the St. Thomas Elgin General Hospital Health Information Management department, which authorized the retrospective review of patient data in accordance with institutional privacy and confidentiality guidelines. This retrospective study was conducted using existing, routinely collected clinical data. A convenience sample of patients was examined before or after the implementation of the novel protocol. Informed consent was waived due to the retrospective nature of the study.

### Protocol for daytime-restricted EN in the ICU

**Figure 1.**
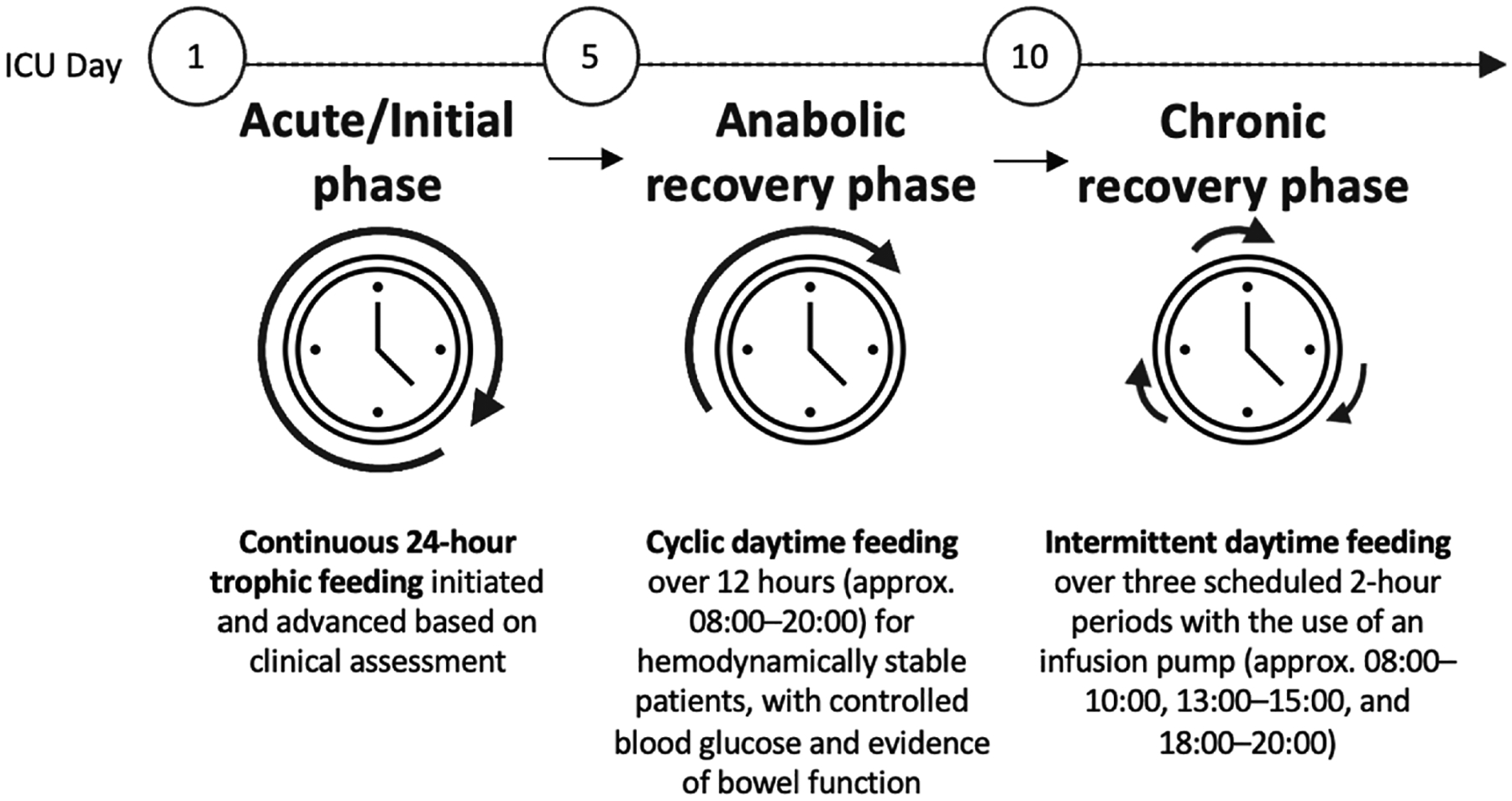
Novel standardized protocol for daytime-restricted enteral nutrition in critically ill adults in the intensive care unit.

The protocol was introduced at the hospital in January 2025 and applied to hemodynamically stable adult ICU patients with functional gastrointestinal tracts. The protocol involved a stepwise transition in EN delivery, beginning with continuous trophic feeding, followed by daytime-restricted 12-hour cyclic feeding, and advancing to intermittent daytime-restricted feeding (described in Figure 1).

On ICU days 1 through 5 (acute/initial phase), trophic feeding was initiated at a continuous rate of 15–20 mL/hr using a standard polymeric formula providing 1.0–1.2 kcal/mL. Continuous trophic feeding was then advanced based on clinical assessment, including gastrointestinal function, vasopressor use, hemodynamic stability, and glycemic control. On ICU days 5 through 10 (or up to 14 based on patient readiness) (anabolic recovery phase), hemodynamically stable patients with controlled blood glucose (e.g., average daily glucose <10 mmol/L) and evidence of bowel function (e.g., bowel sounds or movement, absence of nausea, vomiting, diarrhea, or abdominal distension) were transitioned to cyclic daytime feeding over a 12-hour window (approximately 08:00–20:00). Finally, on ICU days 10 (or up to 14) (chronic recovery phase) and onwards, feeding was provided intermittently during three scheduled 2-hour periods (08:00–10:00, 13:00–15:00, and 18:00–20:00) using an infusion pump, with the infusion rate calculated by dividing the prescribed daily volume equally across the three infusion periods.

Transitions across stages of the protocol were based on individual tolerance, with feeding intolerance defined as nausea, vomiting, diarrhea, or abdominal distension, aligning with the Core Outcome Set for Monitoring of Gastrointestinal Function in Critically Ill Adults (COSMOGI) consensus statement. [14] Adjustments were made by reducing the infusion rate or temporarily pausing feeds.

### Participants

For this retrospective study, critically ill patients requiring EN before and after implementation of the novel protocol were eligible for inclusion. Patients with contraindications were not eligible for the daytime-restricted EN protocol and were therefore excluded from the analysis: extremely high nutritional needs (e.g., severe burns or trauma), hemodynamic instability, known delayed gastric emptying (as evidenced by feeding intolerance, need for post-pyloric feeding, or recurrent gastric retention [14]), recurrent vomiting or ileus, high risk of aspiration, ongoing continuous insulin infusions or uncontrolled hyperglycemia, and recent gastrointestinal surgery; these patients continued to receive continuous feeding and were not advanced to the daytime-restricted 12-hour cyclic feeding schedule.

### Clinical data

Clinical data during ICU admission were retrospectively extracted from patients’ electronic medical records. Relevant data included the number of days and hours per day that EN was administered, the number of hours per day EN was held without provider orders, reasons for EN interruptions, and the type of feeding intolerance documented including the presence of gastrointestinal symptoms such as vomiting, abdominal distension, or constipation leading to the reduction or holding of EN, as documented in the clinical record. Additional data included the actual EN infusion rate (mL/hr), frequency of days EN was held, and the number of days on which goal EN volume was met.

### Statistical analysis

Descriptive statistics for the continuous and daytime-restricted EN groups are presented as medians and interquartile ranges (IQR) for continuous variables given the small sample size and non-normal data distribution, and as frequencies and percentages for categorical variables. Comparisons between the groups were conducted using nonparametric methods. Continuous variables were analyzed using the Mann–Whitney U test, while categorical variables were compared using the chi-square test. The standardized mean differences were reported to quantify the magnitude of between-group differences for continuous variables. A two-sided *P* value < 0.05 was considered statistically significant. All analyses were performed using R (version 4.3.1; R Foundation for Statistical Computing, Vienna, Austria).

## Results

In this preliminary evaluation, a convenience sample of 22 intubated adult ICU patients was examined, including 12 hemodynamically stable patients who received continuous EN prior to protocol implementation (control group) and 10 patients who received daytime-restricted feeding after implementation (Table 1).

The median EN infusion rate in the daytime-restricted group was more than twice that of the continuous group (daytime-restricted: 87.5 mL/hr vs. continuous: 40.0 mL/hr; *P*<0.001), and infusions were delivered over a shorter period each day (daytime-restricted: 11.0 hours/day vs. continuous: 14.5 hours/day; *P*<0.001). Patients in the daytime-restricted group experienced fewer daily interruptions to feeding, with EN infusions held for a median of 1.0 hour/day compared to 9.5 hours/day in the continuous group (*P*<0.001). Additionally, the proportion of uninterrupted feeding days was significantly higher in the daytime-restricted group (daytime-restricted: 76.4% vs. continuous: 55.6%; *P*=0.006). While 90% of the prescribed nutrition was delivered using the daytime-restricted protocol, only 57.5% of the nutritional goal was achieved with continuous infusions (*P*<0.001).

The reasons for EN interruptions did not differ significantly between groups, however constipation was more frequently documented in the continuous group (66.7% vs. 20.0%; *P*=0.079), while vomiting was more frequently observed in the daytime-restricted group (70.0% vs. 41.7%; *P*=0.369).

## Discussion

To our knowledge, this is the first quality improvement study describing the real-world implementation of a standardized daytime-restricted EN feeding protocol in an ICU outside the context of a clinical trial. Overall, findings from the analysis of a small convenience sample of patients suggest that limiting EN delivery to daytime hours may enhance the consistency and adequacy of nutrition provision in ICU adult patients compared to conventional 24-hour continuous feeding. Although no significant differences in gastrointestinal intolerance were observed between the two feeding regimens, further investigations are needed to carefully examine trends noted in the present study.

Patients in the daytime-restricted group received EN at higher infusion rates over a shorter period each day per protocol. The higher infusion rate and condensed feeding window allowed patients to achieve nearly double the prescribed nutritional volume without compromising tolerance. In our study, patients receiving the daytime-restricted EN protocol achieved 90% of the prescribed EN volume, which is considerably higher than the 57.5% achieved in the continuous feeding control group and higher than the proportions reported in previous observational studies of standard continuous EN infusion, which also similarly range from 50 to 60 percent across diverse ICU populations. [15,16].

In our cohort, the daytime-restricted EN group also had fewer feeding interruptions and far fewer hours with EN held compared with the continuous feeding group. The most common reasons for interruptions were vomiting and abdominal distension with daytime-restricted feeding. Earlier multicenter analyses have shown that continuous EN is frequently interrupted for gastrointestinal intolerance or procedures, often resulting in 6–12 hours of lost feeding time per day, which is consistent with the interruptions observed in our continuous feeding group and substantially greater than those in the daytime-restricted group. [6,8,15,16] While vomiting was more common in the daytime-restricted group, there was no indication of associated adverse events or need to revert to continuous feeding. The lower frequency of interruptions observed with daytime-restricted feeding may reflect procedures and extubations being scheduled outside of feeding intervals, consolidation of nutrition delivery within a defined window, or improved gastrointestinal tolerance. However, because data on the timing of procedures and extubations were not available, we cannot confirm this hypothesis. We acknowledge that performing such procedures outside of standard daytime hours may be logistically challenging in some ICUs, and further studies are warranted to examine the feasibility of aligning procedural timing, such as planned extubations, with feeding schedules. Taken together, these preliminary findings in a small sample suggest that daytime-restricted EN may be a feasible and efficacious alternative to continuous 24-hour feeding, complementing existing ICU nutrition strategies such as prokinetics, postpyloric feeding, and semi-recumbent positioning to improve overall nutritional delivery. [17].

Continuous EN feeding remains the standard in critical care, but emerging evidence further supports the feasibility and potential benefits of daytime-restricted EN. [11,12,18] Aligning feeding schedules with the body’s natural circadian rhythms may offer metabolic and gastrointestinal advantages, as continuous feeding has been associated with circadian misalignment, impaired metabolism, disrupted sleep, increased insulin resistance, and reduced autophagy. [19] There is a growing interest in intermittent feeding with studies suggesting comparable efficacy to continuous feeding, [20] and improved whole-body protein synthesis in healthy individuals. [21] Meta-analyses have also shown a lower incidence of constipation with intermittent feeding, [20] possibly due to the preservation of colonic circadian rhythms, which naturally exhibit reduced motility during nighttime hours. [22].

This preliminary study has important limitations. The retrospective study used a small convenience sample of only 22 patients from a single site, which may introduce substantial selection bias and limits generalizability. Data were collected retrospectively from two distinct time periods (Aug–Sep 2023 and Apr–May 2025), which may introduce temporal bias. Extrinsic factors such as seasonal variation or other changes in clinical practice during the 2-year gap period may confound the findings. The sample sizes (12 control, 10 intervention) reflect eligible patients with available data meeting inclusion criteria during each respective 2-month period, however, included participants may not be representative of all eligible patients due to missing data contributing to non-random inclusion. In addition, because of the retrospective study design, patient characteristics beyond sex, such as age, BMI, admitting diagnosis, medical history, and ICU length of stay, were not collected, and other data from existing medical records may be incomplete, representing a major limitation of this study. However, the EN characteristics of the continuous group, as shown in Table 1, are consistent with those reported in previous studies, supporting the representativeness of this sample. [7] Yet, the results and observed trends should be viewed as preliminary and interpreted in the context of the study’s limitations.

## Conclusion

This first real-world evaluation supports the feasibility of implementing a daytime-restricted EN protocol in an adult ICU and highlights its potential in enhancing nutritional adequacy. Broader adoption of this approach should be pursued cautiously and evaluated across diverse patient subgroups. In addition, large prospective studies are needed to validate these results and to examine other clinical outcomes.

## Figures and Tables

**Table 1:** Real-world enteral nutrition implementation metrics comparing a novel daytime-restricted feeding protocol to standard continuous feeding

Variable	Daytime-restricted feeding protocol	Continuous feeding protocol	Standardized mean differences	*P* value
Dates	April–May 2025	August–September 2023		
Patients	10	12		
Sex, Male (%)	7 (70.0)	8 (66.7)		1.00
Days of Data Collection (*n* days)	13.00 [12.00,14.75]	10.50 [9.00, 11.25]	1.13	0.017
Infusion Rate (mL/hr)	87.50 [80.00, 98.75]	40.00 [30.00, 46.25]	3.65	<0.001
Duration of Infusion (hr/day)	11.00 [10.25,12.00]	14.50 [14.00, 18.00]	2.31	<0.001
Duration Infusion Held (hr/day)	1.00 [1.00, 2.00]	9.50 [6.00, 10.00]	3.79	<0.001
Days with any Feeding Interruption (*n* days)	3.00 [2.00, 3.00]	4.00 [3.00, 6.00]	1.04	0.032
Days during Data Collection without any Feeding Interruption (% days)	76.39 [71.25, 83.46]	55.56 [44.09, 67.50]	1.489	0.006
Total Volume Delivered of Prescribed Goal (%)	90.00 [87.50, 92.50]	57.50 [50.50, 58.50]	7.69	<0.001
Reasons for Feeding Interruptions, *n* (%)				
Abdominal Distention	4 (40.0)	5 (41.7)		1.00
Constipation	2 (20.0)	8 (66.7)		0.079
High Gastric Residual Volume	1 (10.0)	3 (25.0)		0.724
Vomiting	7 (70.0)	5 (41.7)		0.369

Continuous variables are reported as median and interquartile range (IQR); categorical variables are presented as number and percentage. Continuous variables were compared using the Mann–Whitney U test and categorical variables with the chi-square test. Standardized mean differences are reported for continuous variables. Abbreviations: hr = hour, mL = milliliter, n = number.

## Data Availability

The data that support the findings of this study are not publicly available due to St. Thomas Elgin General Hospital institutional privacy policies and restrictions on the sharing of patient-level data.
